# Influence of Scanning-Aid Materials on the Accuracy and Time Efficiency of Intraoral Scanners for Full-Arch Digital Scanning: An In Vitro Study

**DOI:** 10.3390/ma14092340

**Published:** 2021-04-30

**Authors:** Hyun-Su Oh, Young-Jun Lim, Bongju Kim, Myung-Joo Kim, Ho-Beom Kwon, Yeon-Wha Baek

**Affiliations:** 1 Department of Prosthodontics and Dental Research Institute, School of Dentistry, Seoul National University, Seoul 03080, Korea; hsoh1391@snu.ac.kr (H.-S.O.); silk1@snu.ac.kr (M.-J.K.); proskwon@snu.ac.kr (H.-B.K.); 2 Dental Life Science Research Institute, Seoul National University Dental Hospital, Seoul 03080, Korea; 3 Department of Prosthodontics, Seoul National University Gwanak Dental Hospital, School of Dentistry, Seoul National University, Seoul 03080, Korea; notus@hanmail.net

**Keywords:** intraoral scanners, full arch, trueness, precision, time efficiency, scanning-aid materials

## Abstract

This study was performed to verify the influence of scanning-aid materials on the accuracy and time efficiency of full-arch scanning with intraoral scanners. The full-arch reference model was constructed by a 3D printer and scanned with a model scanner to obtain the reference dataset. Four experimental groups (application of ScanCure (SC-80, ODS Co, Incheon, Korea), IP Scan Spray (IP-Division, Haimhausen, Germany) and Vita Powder Scan Spray (Vita Zahnfabrik, Stuttgart, Germany), and no treatment) were designed, and the scans were executed (trueness, n = 5) using two intraoral scanners: I500 (Medit Co., Seoul, Korea) and TRIOS (3shape, Copenhagen, Denmark). All acquired scan data were compared with the reference datasets using the 3D superimposition method and 2D linear measurements. In the 3D analysis, intragroup data were compared with each other (precision, n = 10). Time efficiency was also verified by comparing the scan times of the four experimental groups. In the 3D analysis, the root mean square (RMS) value of the precision of the scanned image was statistically significantly more accurate in the scanning-aid agent-treated groups than in the no-treatment group *(p* < 0.05). However, the RMS values of trueness and the types of scanning-aid materials were not significantly different. In the 2D measurements, the increased scan distance generated a greater distance deviation. The working time was significantly shorter in the scanning-aid agent groups than in the no-treatment group, with statistical significance (*p* < 0.05). Therefore, in clinical situations, the application of scanning-aid materials is recommended to reduce scanning time and more efficiently obtain the full-arch scanned image.

## 1. Introduction

Recently, with the development of computer-aided design (CAD) and computer-aided manufacturing (CAM) systems, several commercial intraoral scanners with high accuracy have been introduced to the market and are increasingly being used in clinical practice for intraoral digital scanning. The main concern with the intraoral scanner systems (IOS) is enhancing their accuracy. Several studies have reported that the current IOS yields less deviation or even higher accuracy compared with conventional impressions for short spans limited up to a quadrant or three abutments [1,2,3,4].

In orthodontic treatment, intraoral scanners may be a useful alternative for full-arch scans for diagnostic purposes [5]. However, for prosthodontic use, the accuracy of marginal and internal adaptation for the passive fit of prostheses is important for the success of the long-term prognosis of prostheses [6]. Therefore, to use IOS clinically in fabricating prostheses for full-arch cases, the accuracy of IOS over a long span should be evaluated and verified.

Many studies have verified several scanners proposed for complete-arch digital impression in clinical situations for accuracy, time efficiency, and patient convenience [7,8,9,10]. In full-arch implant rehabilitation, Amin et al. [11] reported that full-arch digital implant impressions using the 3M True Definition and Cerec Omnicam were significantly more accurate than the conventional impression with the splinted open-tray techniques.

In contrast, other studies have reported that intraoral scanners experience difficulties in scanning complete dental arches or edentulous arches with multiple implants and constructing precise virtual images [12,13,14,15]. This is because the risk of error in accuracy can be affected as the scan area increases [12]. Whether the full arch application satisfies the level of accuracy needed for clinical implementation remains to be investigated [15].

To minimize the unwanted background noise and scanning error caused by artificial reflective surfaces such as metallic materials, titanium dioxide powder should be applied to the object to be scanned [16]. However, when the operators apply powder-type agents to the teeth, different application distances and times result in different thicknesses of the coating layer, which causes scan errors and respiratory problems from scattered particles [17,18]. Liquid-type scanning-aid agents with brush techniques do not have these disadvantages [19]. There are powder-free type intraoral scanners, but these still face difficulties in optical properties caused by various oral environment factors (saliva, blood, metallic surfaces, etc.) [20,21]. Some studies reported that the accuracy of powder-free scanners can be improved by using a powder coating [22].

The aim of this study was to assess the accuracy of two different intraoral scanners on a specially designed full-arch reference model with several scanning-aid materials. To evaluate the accuracy, 3D superimposition with best-fit alignment and linear measurements of the designated distances on the reference model were used. Additionally, the time efficiency was verified by measuring the scan time. Therefore, the effectiveness of each scanning-aid material for two intraoral scanners was compared and assessed.

## 2. Materials and Methods

### 2.1. Reference Model

**Figure 1:**
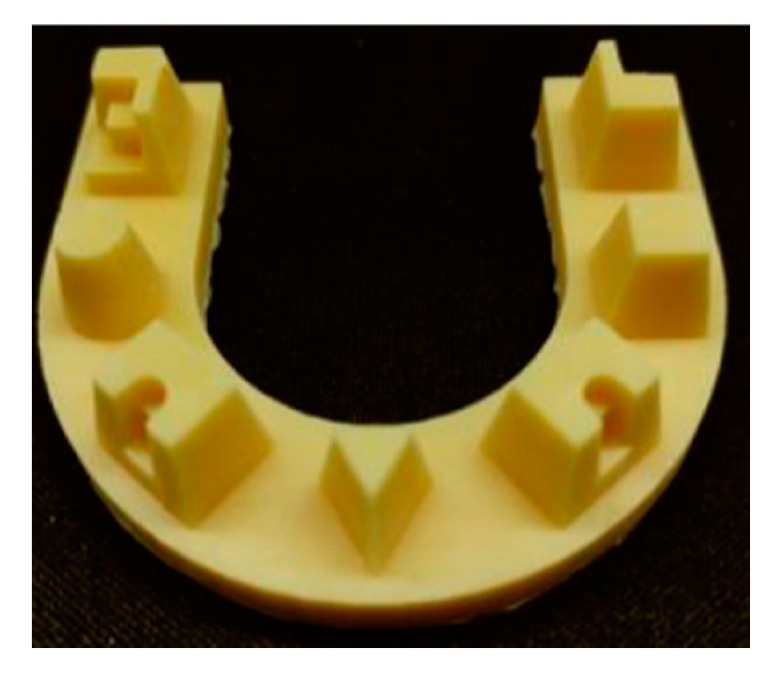
Full-arch reference model fabricated by a 3D printer.

The full-arch reference model was designed using a CAD program (Solidworks 2016, Dassault Systèmes SolidWorks Corp., Waltham, MA, USA) and was fabricated by a 3D printer (Perfactory Micro 3D Printer, EnvisionTec, Dearborn, MI, USA) (Figure 1). The material for the printed reference model was E-Denstone (EnvisionTec, Dearborn, MI, USA). This reference model was fabricated to evaluate the accuracy of the digital impression obtained by two intraoral scanners with several scanning-aid agents.

The full-arch reference model was designed asymmetrically to reflect the situations in which the intraoral scanner experienced recognition difficulty due to the mirror image when constructed symmetrically; it was composed of two inlay forms, two onlay forms, and three different crowns. Shapes were placed in one parabolic arch base to simulate the full dental-arch restoration (Figure 1).

### 2.2. Digital Impressions

A reference dataset was obtained by scanning the reference model with laboratory scanner (Identica Hybrid, Medit Co., Seoul, Korea). The model was scanned with three different types of scanning-aid materials using the following two different intraoral scanners: I500 (Medit Co., Seoul, Korea) and TRIOS (3shape, Copenhagen, Denmark). The scanning procedure using the intraoral scanners was repeated five times (trueness, n = 5) for each experimental group (ScanCure, IP Scan Spray, Vita Powder Scan Spray, and no treatment). The scanned datasets were exported into standard tessellation language (STL) data formats. All scanned STL files were compared with the reference file (trueness, n = 5), and intragroup data were compared with each other (precision, n = 10). In each cycle of digital impression, the scanning-aid materials on the surface of the model were removed by organic solvents and an air compressor water gun to clean the model. The scanning procedure was executed by one proficient prosthodontist, following the manufacturer’s instructions.

### 2.3. Scanning-Aid Materials

In this experiment, three different kinds of scanning-aid materials were used: ScanCure (SC-80, ODS Co., Incheon, Korea), Vita Powder Scan Spray (Vita Zahnfabrik, Stuttgart, Germany), and IP Scan Spray (IP-Division, Haimhausen, Germany). ScanCure is a liquid-type scanning-aid agent that was applied by the brush technique, while the others were powder-type scanning-aid agents applied by the spraying technique. The color of the ScanCure and IP is white, and the VITA has a light blue color.

### 2.4. Three-Dimensional Measurements

The acquired STL datasets were used to verify the trueness and precision of the four experimental groups with two intraoral scanners. All STL datasets were loaded into 3D comparison software (Geomagic Control X, 3D systems, Rock Hill, SC, USA) and superimposed with the reference file using the best-fit algorithm. The best-fit algorithm is the function of 3D analysis software for correcting the position to generate minimum error between STL datasets to be compared. Based on least square regression, the alignment was performed to minimize the error with a set tolerance of ±0.015 mm and a maximum tolerance range of ±0.3 mm. The mean and standard deviation of the experimental groups was assessed using the root mean square (RMS) value.

### 2.5. Two-Dimensional Measurements

**Figure 2:**
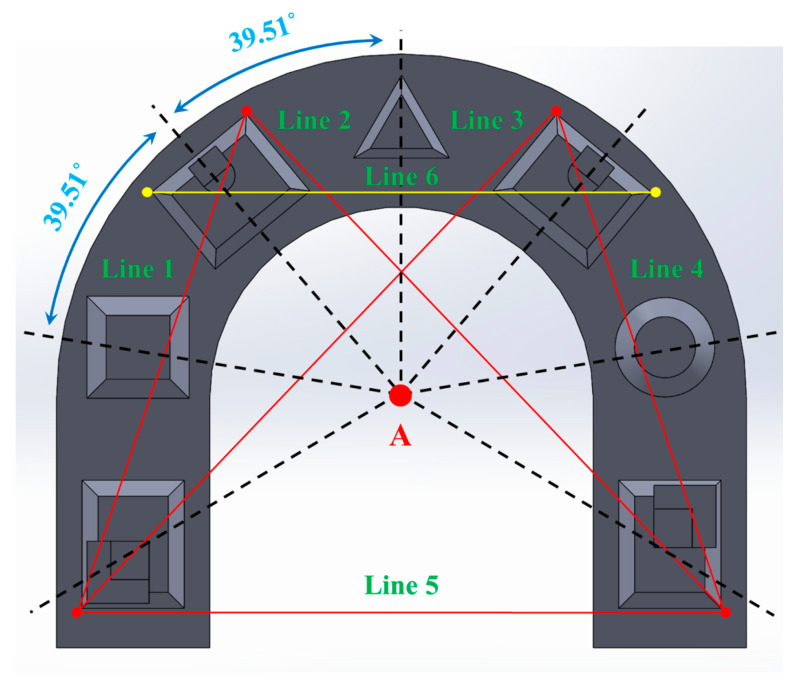
Six points and the linear measurement parameters (lines 1 to 6) between each point in the full-arch reference model. Line 1: Distance between left red points. Lines 2, 3: Distance of diagonal line between left red point and right red point. Line 4: Distance between right red points. Line 5: Distance of the posterior horizontal line between two red points. Line 6: Distance of the anterior horizontal line between two yellow points.

For 2D comparison of the reference model, six designated points and lines on the model were used. Scanned images were located in the same position, and six linear measurement parameters were calculated using the Rhinoceros 5 software (Robert McNeel & Associates, Seattle, WA, USA). Six parameters were acquired in each scan image, and the mean and standard deviation of each parameter were assessed. The designated lines 1 to 6 are defined below (Figure 2).

### 2.6. Scanning Time Measurements

To evaluate and compare the time efficiency of scanning-aid materials, the time to scan the full-arch reference model in the four experimental groups was measured using two intraoral scanners.

### 2.7. Statistical Analysis

Statistical analysis was performed by one-way ANOVA multiple comparisons (SigmaPlot 14.0, Systat Software Inc., San Jose, CA, USA). Multiple comparisons were performed according to the Student-Newman-Keul method. Statistical significance was set at *p* < 0.05.

## 3. Results

### 3.1. Three-Dimensional Analysis

#### 3.1.1. Trueness

**Figure 3:**
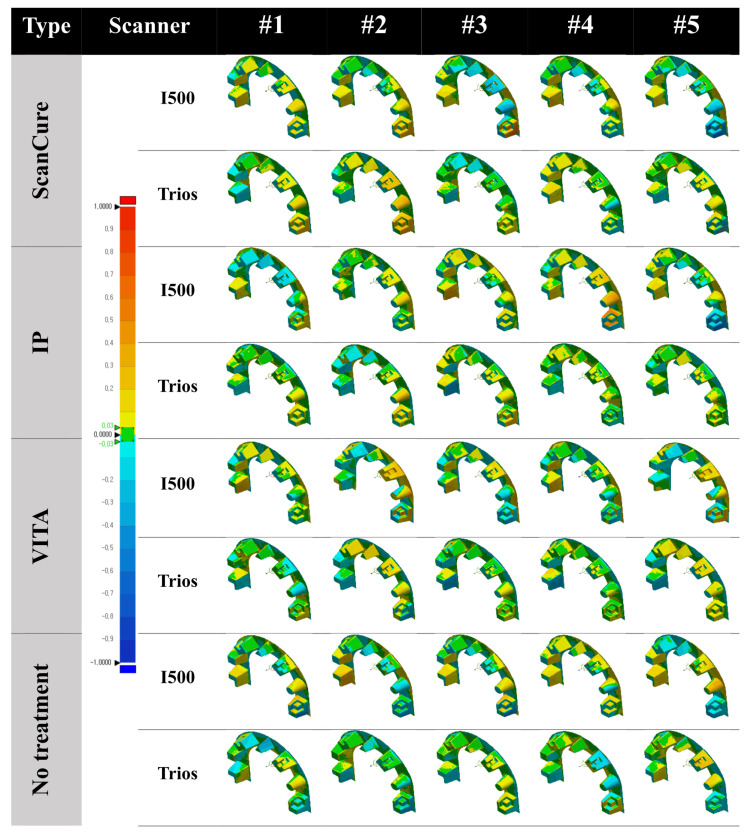
Superimposed error distribution in the full-arch reference model with four experimental groups using two intraoral scanners (I500 and Trios) (tolerance range ±30 μm).

When the error tolerance range was set to 30 μm, all experimental groups showed similar errors in distribution with both scanners (Figure 3).

**Figure 4:**
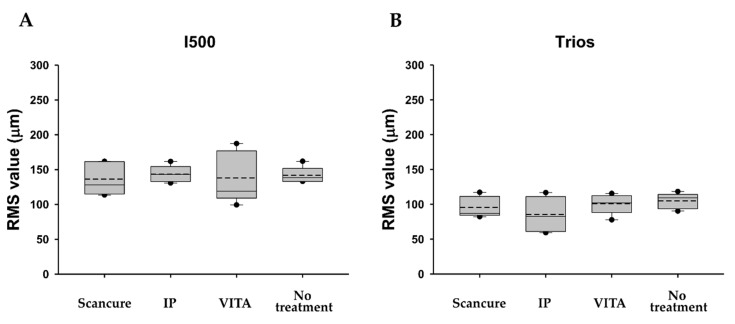
Box plots of the RMS values (trueness) for the four experimental groups (ScanCure, IP, VITA, no treatment) with the two intraoral scanners. (**A**) I500 and (**B**) Trios. RMS, root mean square.

With the I500 scanner, the mean error of the ScanCure group was smaller than that of the other groups. With the Trios scanner, the mean error of the IP group was the smallest among the experimental groups. However, there were no statistically significant differences among the four experimental groups with the one-way ANOVA test including both scanners (*p* = 0.909 and *p* = 0.348, respectively) (Table 1, Figure 4).

When comparing errors with two different intraoral scanners (I500, Trios), all experimental groups (ScanCure, IP, VITA, no treatment) showed statistically significant differences. In the ScanCure group, the error of the Trios scanner was 40.50 μm less than that of the I500 scanner, with a 95% statistical significance (*p* = 0.02). In the IP group, the error of the Trios scanner was 58.04 μm less than that of the I500 scanner (*p* = 0.002). In the VITA group, the error of the Trios scanner was 37.40 μm less than that of the I500 scanner (*p* = 0.035). In the no-treatment group, the error of the Trios scanner was 36.68 μm less than that of the I500 scanner (*p* = 0.04). We confirmed that the I500 scanner showed a higher error than the Trios scanner in the full-arch reference model for all experimental groups.

#### 3.1.2. Precision

**Figure 5:**
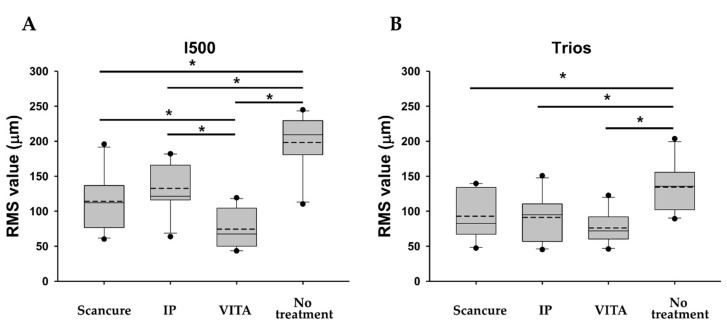
Box plots of the RMS values (precision) for the four experimental groups (ScanCure, IP, VITA, and no treatment) with the two intraoral scanners. (**A**) I500 and (**B**) Trios. RMS, root mean square.

**Figure 6:**
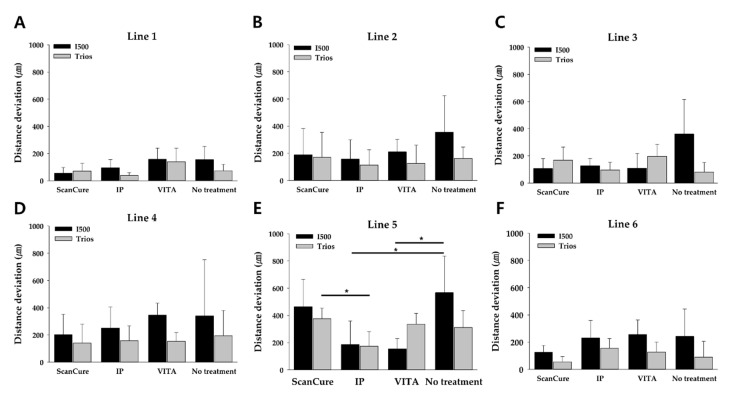
Bar graphs of the mean distance deviation of lines 1–6 (**A**–**F**) for the four experimental groups with the two intraoral scanners.

In the I500 scanner, the no-treatment group showed a statistically significantly higher RMS value than each agent-applied group (ScanCure, IP, VITA) (*p* < 0.05) (Figure 5A). The VITA group showed a statistically significantly lower RMS value than the ScanCure and IP groups (*p* = 0.022 and *p* = 0.004, respectively) (Figure 6A). In the Trios scanner, the no-treatment group also had a higher RMS value than each agent-applied group (ScanCure, IP, VITA) with a statistically significant difference (*p* < 0.05) (Figure 5B). When comparing the two intraoral scanners, the Trios scanner had a statistically significantly lower RMS value than the I500 scanner in the IP and no-treatment groups (*p* < 0.05) (Table 2).

### 3.2. Two-Dimensional Analysis

We measured the distance deviations of the six lines that were defined by connecting the six designated points on the full-arch model to verify the accuracy of shape reproducibility in the full-arch model (Figure 2). When the model was scanned by the I500 scanner, there were statistically significant differences at the 95% significance level in line 5; the distance deviation of the no-treatment group was the longest; and the distance deviations of the IP and VITA groups were 381.02 and 412.36 μm, respectively, which was shorter than the no-treatment group (*p* = 0.017 and *p* = 0.018, respectively) (Table 3, Figure 6). In lines 1–4 and 6, there were no statistically significant differences among the groups (*p* > 0.05). In lines 1, 3, 4, and 6, the ScanCure group had a lower distance deviation than the other groups (Table 3, Figure 6).

When the model was scanned by the Trios scanner, there were also statistically significant differences at the 95% significance level in line 5; the distance deviation of the IP group was the shortest, and the distance deviation of the ScanCure group was 202.40 μm longer than that of the IP group with statistical significance (*p* = 0.024). Except for line 5, in lines 1–4 and 6, there were no statistically significant differences among the groups (*p* > 0.05). In lines 1, 2, 3, and 5, the IP group had a lower distance deviation than those of the other groups (Table 3, Figure 6).

### 3.3. Scanning Time Analysis

**Figure 7:**
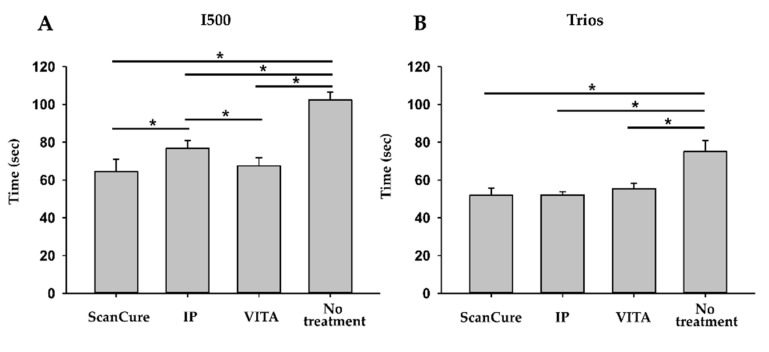
Bar graph of the scanning times of the four experimental groups (ScanCure, IP, VITA, and no treatment) with the two intraoral scanners. (**A**) I500, (**B**) Trios.

In the I500 scanner, the scanning time of scanning-aid material-applied experimental groups (ScanCure, IP, VITA) was shorter than that of the no-treatment group (*p* < 0.001, Table 4, Figure 7A). In addition, the ScanCure and VITA groups had shorter scanning times than the IP group (*p* = 0.003 and *p* = 0.008, respectively; Table 4, Figure 7A). In the Trios scanner, all groups with scanning-aid material had a shorter scanning time than the no-treatment group (*p* < 0.001; Table 4, Figure 7B). However, there were no statistically significant differences among the scanning-aid material-applied groups (ScanCure, IP, VITA) (*p* > 0.05; Table 3, Figure 7B). Comparing the two intraoral scanners, the Trios scanner had a significantly shorter scanning time than the I500 scanner for all experimental groups (*p* < 0.001).

## 4. Discussion

In our previous study, which was performed with inlay, onlay, and bridge reference models, the scanning-aid material-applied groups had significantly better accuracy than the no-treatment group, and the liquid-type scanning-aid material had superior shape reproducibility compared with other powder-type materials [19]. However, in this study with the full-arch reference model, there were no statistically significant differences (trueness, n = 5) among the experimental groups (ScanCure, IP, VITA, and no treatment) in the 3D analysis. It was thought that the increased scan distance caused a large error in the RMS value (trueness, n = 5) by accumulating the volumetric data. The primary reason for the enhanced errors on longer scans, such as full-arch scans, may be matching or stitching errors, which increase with lengthening of the scan [23]. Intraoral scanners cannot scan the entire arch in one image but instead obtain single images that are stitched with other images to construct a digital 3D model of the object being scanned. This stitching process with the best-fit software algorithm can produce errors that are proportional to the scan distance.

In comparison with the intraoral scanners, the Trios scanner showed better shape reproducibility than the I500 in all experimental groups (*p* < 0.05). Especially in full-arch scanning, not only the type of scanner but also the scanning protocol, such as the scanning path, affects the accuracy of digital scanning [24]. The operator’s factors, such as scanning skills and learning curves, also affect the accuracy of scans [25]. In this study, one experienced prosthodontist performed the scans following the manufacturer’s recommended strategy to avoid errors from different scanning styles.

Considering the accumulated volumetric errors of the 3D analysis, 2D analysis was performed to minimize the RMS error arising from 3D superimposition. However, along with the results of the 3D analysis, no significant differences in distance deviation (µm) were measured among experimental groups, except for line 5. Line 5 is the horizontal line, which was formed by connecting two points wherein the scan started and finished. Therefore, the differences may have originated from the increased scan distance, as mentioned above.

Contrary to our expectation, the result of scanning accuracy between the applied and non-applied groups was not statistically significant. Not only the increased scan distance in the full-arch scan, but also the limitation in simulating the real oral environment, such as a metallic crown, was considered as the reason for the lack of significant differences among groups. Scanning-aid materials are generally composed of titanium dioxide to enhance the opacity of the surface and enable the uniform reflection of light. Therefore, scanning-aid materials can be more effective on metallic surfaces than resin-like materials because they reduce the reflections that prevent the scanners from recognizing the objects to be scanned.

Similar to our previous study [19], the scanning time of the applied groups was significantly shorter than that of the no-treatment group with both scanners (*p* < 0.05).

These results can be explained by the scanning material creating a uniform reflective surface and enhancing the opacity of scanned objects so intraoral scanners can recognize them more efficiently [26]. However, scanning time is affected by not only the scanner’s quality of recognition but also the limited situations of patients (saliva, tongue, trismus, and patient’s uncontrolled movement, etc.) and the proficiency of the operator [25]. Therefore, it is considered that the application of scanning agents is more efficient in scanning the full arch than no treatment with agents in real clinical situations.

The limitation of the present study was that the designed full-arch reference model did not simulate the real size of the patient’s full dental arch and various intraoral environments, such as the presence of saliva, blood, and especially the reflective properties of metallic prostheses. In order to verify the efficiency and accuracy of scanning agents in real clinical situations, such as metallic prostheses, further studies with a model simulating the reflective properties of metal are necessary.

The clinical implications of this study are, when applying the scanning-aid agents to the full dental arch, that the time efficiency and precision of the scanned data are significantly better than those without treatment. These findings suggest that scanning-aid materials can be applied to efficiently obtain full-arch scan data in real clinical situations.

## 5. Conclusions

Within the limitations of the in vitro resin model study, the following conclusions were drawn:

1. In the 3D analysis, the RMS values (precision, n = 10) of the scanning-aid material-applied groups were significantly lower than those of the no-treatment group. The application of scanning-aid materials might affect the precision of scanned data rather than trueness.
2. In the 2D analysis, the longest scan distance, line 5, showed the largest distance deviation, which meant that the scanned data were less accurate as the scan distance was longer, as in the full-arch case.
3. When the scanning-aid agents were applied in the full-arch model, the scanning time was shortened compared with the no-treatment group, with a statistically significant difference.
4. Consequently, in real clinical environments with limitations such as intraoral saliva, tongue, etc., the application of scanning-aid materials can reduce working time and more efficiently obtain the full-arch scanned image.

## Figures and Tables

**Table 1:** Mean RMS values of trueness for the four experimental groups with the two intraoral scanners (mean ± SD).

Trueness	ScanCure (µm)	IP (µm)	VITA (µm)	No Treatment (µm)
I500	136.18 ± 23.69	143.48 ± 11.99	138.18 ± 36.98	141.70 ± 11.89
Trios	95.68 ± 15.03	85.44 ± 25.38	100.78 ± 14.43	105.02 ± 11.25

SD, standard deviation. RMS, root mean square.

**Table 2:** Mean RMS values of precision for the four experimental groups with the two intraoral scanners (mean ± SD).

Precision	ScanCure (µm)	IP (µm)	VITA (µm)	No Treatment (µm)
I500	114 ± 40.8	133 ± 35.7	74.3 ± 27.9	198 ± 42.5
Trios	92.9 ± 34.2	91.3 ± 32.5	76.1 ± 22.4	134 ± 34.7

SD, standard deviation. RMS, root mean square.

**Table 3:** The mean distance deviation (μm) of lines 1 to 6 between the four experimental groups using two intraoral scanners in the full-arch reference model.

Type			ScanCure	IP	VITA	No Treatment
**Line 1**	I500	Avg.	55.16	95.40	157.58	154.88
		S.D.	41.83	59.57	83.84	98.28
	Trios	Avg.	70.66	38.84	139.56	72.54
		S.D.	57.94	21.05	100.97	48.36
**Line 2**	I500	Avg.	188.58	157.20	211.78	355.84
		S.D.	193.26	142.96	91.67	269.72
	Trios	Avg.	170.84	113.60	125.96	162.44
		S.D.	183.41	112.84	134.07	83.44
**Line 3**	I500	Avg.	107.06	126.94	109.98	360.26
		S.D.	72.93	54.42	107.71	254.92
	Trios	Avg.	169.28	96.18	197.40	81.08
		S.D.	96.01	57.43	88.87	69.84
**Line 4**	I500	Avg.	201.56	251.38	345.20	340.66
		S.D.	150.86	154.03	88.44	411.30
	Trios	Avg.	140.24	158.06	152.54	194.90
		S.D.	139.90	109.60	64.20	185.61
**Line 5**	I500	Avg.	463.60	186.08	154.74	567.10
		S.D.	202.13	174.05	74.49	268.21
	Trios	Avg.	376.74	174.34	336.56	312.26
		S.D.	76.91	106.81	79.00	124.06
**Line 6**	I500	Avg.	125.24	230.44	257.00	242.12
		S.D.	50.77	129.20	105.14	200.91
	Trios	Avg.	55.52	155.46	127.18	90.02
		S.D.	38.28	71.14	73.14	115.53

**Table 4:** Working times of the four experimental groups (ScanCure, IP, VITA, and no treatment) with the two intraoral scanners (I500 and Trios) (mean ± SD).

Time	n	ScanCure (s)	IP (s)	VITA (s)	No Treatment (s)
I500	5	64.55 ± 5.75	76.75 ± 3.65	67.48 ± 3.81	102.37 ± 3.76
Trios	5	51.86 ± 3.40	52.39 ± 1.63	55.43 ± 2.60	75.03 ± 5.19

SD, standard deviation.

## Data Availability

The data presented in this study are available on request from the corresponding author.
